# Epidemiology of Progressive Supranuclear Palsy: Real World Data from the Second Largest Health Plan in Israel

**DOI:** 10.3390/brainsci12091126

**Published:** 2022-08-24

**Authors:** Yael Barer, Gabriel Chodick, Raanan Cohen, Meital Grabarnik-John, Xiaolan Ye, Jorge Zamudio, Tanya Gurevich

**Affiliations:** 1 Maccabitech, Maccabi Institute for Research and Innovation, Tel Aviv-Yafo 6812509, Israel; 2 Sackler School of Medicine, Tel Aviv University, Tel Aviv-Yafo 6997801, Israel; 3 AbbVie Inc., Hod Hasharon 4511001, Israel; 4 AbbVie Inc., North Chicago, IL 60064, USA; 5 Tel Aviv Sourasky Medical Center, Tel Aviv-Yafo 6423906, Israel; 6 Sagol School of Neuroscience, Tel Aviv University, Tel Aviv-Yafo 6997801, Israel

**Keywords:** progressive supranuclear palsy, prevalence, incidence, clinical features, real world data, natural history, future disease modifying trials

## Abstract

Progressive supranuclear palsy (PSP) is a rare and fatal neurodegenerative movement disorder and no disease modifying therapy (DMT) is currently available. This study aims to assess the epidemiology of PSP in Israel and to describe its clinical features. This retrospective analysis identified patients with PSP between 2000 and 2018 over the age of 40 years at first diagnosis (index date). We identified 209 patients with ≥1 diagnosis of PSP. Of those, 88 patients satisfied the inclusion criteria with a mean age at diagnosis of 72 years (SD = 8) and 53% were female. The 2018 prevalence and incidence rates were 5.3 and 1 per 100,000 persons, respectively. Median survival time was 4.9 years (95% CI 3.6–6.1) and median time from initial symptom to diagnosis was 4.2 years. The most common misdiagnoses were Parkinson’s disease, cognitive disorder and depression. The present study demonstrates that the clinic-epidemiological features of PSP in Israel are similar to PSP worldwide. In light of PSP’s rarity, investigation of PSP cohorts in different countries may create a proper platform for upcoming DMT trials.

## 1. Introduction

Progressive supranuclear palsy (PSP) is a rare neurodegenerative movement disorder, with an estimated annual prevalence of 5–7 per 100,000 persons [1,2] and annual incidence density rate between 0.9 and 2.6 per 100,000 persons [3,4], which both increase with age [5]. The classic phenotype, identified in 1963, referred to now as PSP-RS (Richardson’s syndrome), presents with postural instability and axial rigidity, leading to falls, supranuclear gaze palsy, frontal-subcortical cognitive impairment, and dysphagia, leading to aspiration [6]. Although most patients eventually develop the majority of PSP-RS’s features, two-thirds of patients demonstrate other clinical variants, with PSP-Parkinsonism (PSP-P) as the second most common phenotype [7], characterized by different initial symptoms, such as more tremors and less falls [7,8], leading to a delayed diagnosis by 3–4 years [9]. Once diagnosed, PSP is a fatal disease where the median survival time ranges between 5 and 8 years, but differs between PSP phenotypes, with shorter life expectancy in patients with RS phenotypes as compared to non RS phenotypes [6,10]. While PSP’s fatality rises, there are no approved disease modifying therapies (DMT) [11,12]. Furthermore, satisfying symptomatic treatment is also lacking. While some patients experience benefits from the use of levodopa, these benefits are transient and have no effect on disease duration [8].

To the best of our knowledge, in Israel, there are no publications that describe the epidemiology and clinical features of PSP. Therefore, this study aims to assess the prevalence and incidence of PSP in Israel and to describe the clinical features of the disease, including initial symptoms, common misdiagnoses, survival time and treatment patterns.

## 2. Materials and Methods

### 2.1. Study Design and Patients

This retrospective analysis was performed on Maccabi healthcare services’ (MHS) automatically computerized clinical database. MHS is a nationwide health plan (payer-provider), representing a quarter of the population in Israel (~2.6 million people) with a <1% per year moving out rate. The National Health Insurance Law (NHIL), accepted in Israel in 1995, mandated that all legal residents were required to enroll in one of the four not for-profit health plans and are guaranteed free choice among them [13].

We selected all patients with PSP (PwPSP) who were diagnosed between 2000 and 2018 (inclusive) over the age of 40 years at first diagnosis. Patients were identified by *The International Classification of Diseases, Ninth Edition* (ICD-9) 333.0 (“other degenerative diseases of the basal ganglia”), given by hospitals or MHS internal diagnosis code for PSP. Due to its unspecific nature, potential cases indicated solely by ICD-9 333.0 were verified by reviewing hospital discharge reports. First diagnosis was defined as the index date.

Annual prevalence was assessed for PSP in 2018. All MHS members aged ≥40 years old at diagnosis who were alive and active in MHS in mid-2018 were included in the denominator (N = 920,112). PwPSP aged ≥40 years’ old who were alive and active in MHS during 2018 comprised the numerator. Annual incidence of PSP was calculated for 2018 among MHS members aged ≥40 years old with at least 1 year of continuous enrolment (N = 912,721).

### 2.2. PSP Clinical Features

PSP clinical features (i.e., symptoms, comorbidities, treatment patterns and misdiagnoses) were evaluated among patients with at least 12 months of membership in MHS prior and post index.

### 2.3. Variables and Measurements

Crude and age-adjusted prevalence and incidence rates were assessed. Patients’ demographics at index date included age, sex, smoking status and socioeconomic status (SES). SES was based on a score ranked from 1 (lowest) to 10, derived for commercial purposes by Points Location Intelligence, using geographic information systems (GIS) and data, such as expenditures related to retail chains, credit cards and housing. This score is highly correlated with SES measured by the Israel Central Bureau of Statistics [14]. SES was categorized into low (1–4), medium (5–6) and high (7–10).

### 2.4. Symptoms, Comorbidities and Misdiagnoses

PSP clinical features were described by (1) initial and most common symptoms; (2) common misdiagnoses; (3) chronic comorbidities; (4) time to disability from index. Disability was defined by Israeli social security codes (see list of codes in Table A1); and (5) treatment patterns.

Physician diagnoses during a five year-period prior index were extracted and categorized into symptoms, comorbidities and misdiagnoses. The most common diagnoses in each group were explored (Table A2, Table A3 and Table A4). Data were also extracted from MHS validated chronic disease registries; cardiovascular disease (CVD, including cerebrovascular disease) [15], diabetes [16], hypertension [17], chronic kidney disease (CKD) [18] and cancer [19]. Initial symptoms and misdiagnoses were assessed in the five years preceding the index date. Newly diagnosed chronic comorbidities were assessed ever prior and post index date. For the first symptom, a sensitivity analysis was preformed, excluding the symptom “pain”, due to its lack of specificity.

### 2.5. Treatment Patterns

Data on dispensed medications were extracted for a period of one year before and after the index date. A more detailed exploration was performed for the anti-Parkinsonian pharmacologic medications, where purchases were categorized by generic ingredients. Patients were considered under the treatment of a medication if they had at least one purchase.

### 2.6. Statistical Methods

The study was conducted in accordance with the ICH-GCP, Good Epidemiology Practices (GEP) and Declaration of Helsinki, Ethical Principles for Medical Research Involving Human Subjects. A waiver of consent was approved by the Assuta Medical Centers ethics committee. Crude and age-specific rates were calculated to assess the 2018 annual prevalence and incidence per 100,000 MHS members over the age of 40 years. Adjusted rates were calculated using the WHO standard population [20].

**Figure 1:**
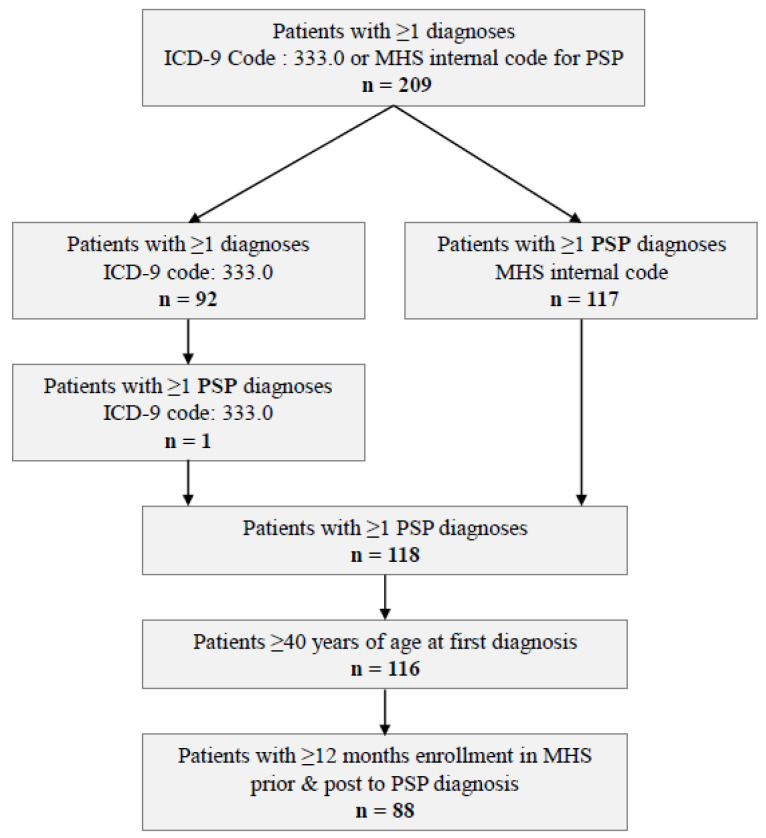
Attrition process of patients with PSP.

A comparison of age-specific rates was carried out to recent publications. Rates from Viscidi et al. [4] were extracted from Figure 1. The exact binomial 95% confidence intervals (95% CI) were calculated using the reported number of cases to estimate the populations’ sizes in each age group. The numbers, reported in ten years’ gaps, were divided into two equal parts. For Coyle-Glichrist et al. [21], exact rates were extracted from Figure 1, using “Microsoft Publisher”. Population size in each age group was calculated using the reported population size (1.69 million) and the 2013 Europe standard population [22]. Descriptive statistics are presented using frequencies and proportions for the categorical variables and mean values with standard deviation (SD) or medians with interquartile range (IQR) as appropriate. Time to event was calculated and presented using Kaplan–Meier curves. All analyses were performed in SPSS 25 (IBM). Figures were created using R software.

## 3. Results

We identified 209 patients with at least 1 diagnosis of PSP or “other degenerative diseases of the basal ganglia”. Of those, 117 were diagnosed at least once by the PSP-specific MHS internal code. Ninety-two patients were diagnosed with only “other degenerative diseases of the basal ganglia” ICD-9 code; of those, only one patient was truly diagnosed with PSP. After excluding patients diagnosed before the age of 40, the PSP cohort included 116 patients (Figure 1).

### 3.1. Prevalence, Incidence and Survival

**Figure 2:**
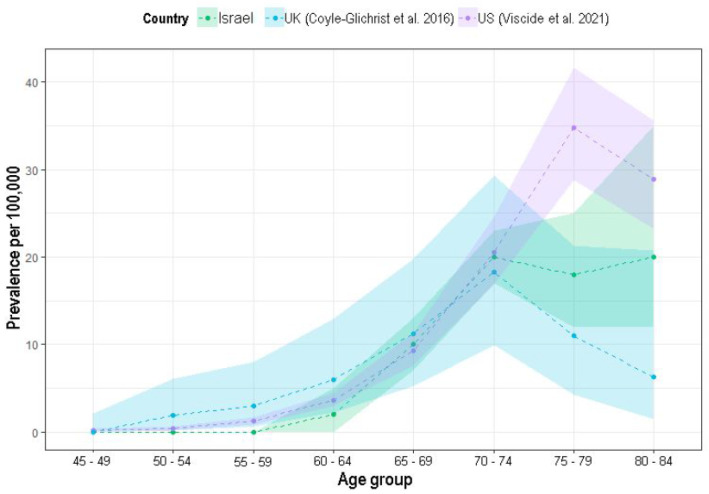
PSP age-specific prevalence rates in 2018. [4,21]

**Figure 3:**
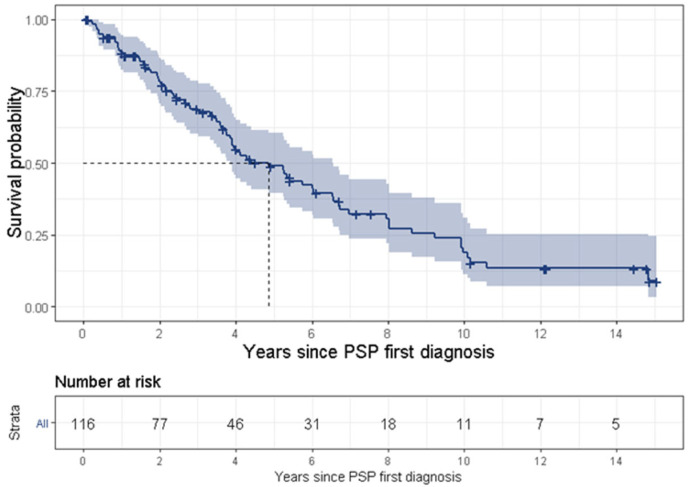
Survival curve of patients with PSP, from first diagnosis, calculated using Kaplan–Meier curve.

The crude (over the age of 40 years) and the age-adjusted prevalence rates in 2018 were 5.3 per 100,000 MHS members and 1.6 per 100,000 persons, respectively. Figure 2 presents the age-specific prevalence rates compared to more recent publications [4,21]. The crude (over the age of 40 years) and age-adjusted incidence rates for 2018 were 1 per 100,000 MHS members and 0.29 per 100,000 persons, respectively. The median survival time was 4.9 years (95% CI 3.6–6.1) post diagnosis and 23 patients (19.8%, 95% CI: 13.3–27.8%) of patients had overall survival rates of more than 10 years (Figure 3).

### 3.2. Patients with PSP Characteristics

For the 88 patients who satisfied inclusion criteria, the mean age of diagnosis was 72 years (SD = 8). A total of 53% were female and 6.8% (8.6% of those with smoking data) of patients were past or current smokers (Table 1). Ninety-two percent of patients had more than five years’ continuous membership in the MHS prior index. 

### 3.3. PSP Clinical Features

**Figure 4:**
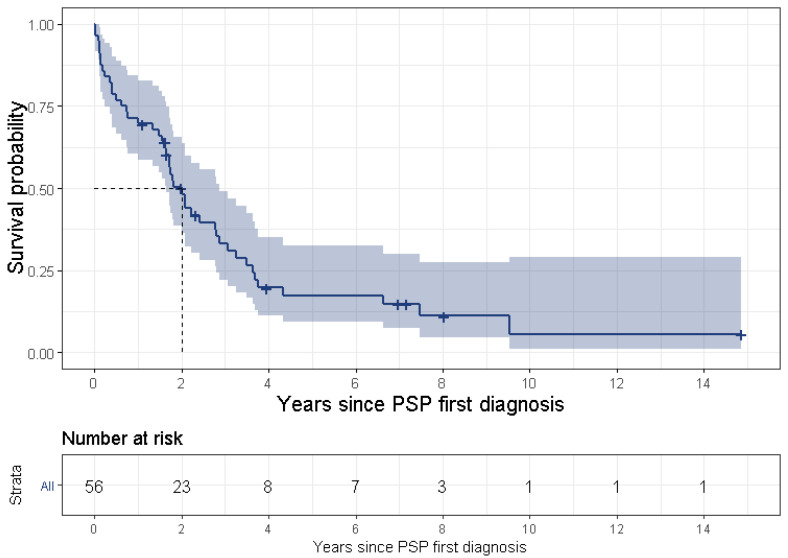
Time to disability, PwPSP with no disability at baseline (n = 56), calculated using Kaplan–Meier curves.

The median time from initial symptom to first diagnosis was 4.2 years (IQR: 3.5–4.8 years) and the most common first symptoms were pain (52%), vertigo (15%) cough and gait abnormalities (both with 7%). Symptoms with a frequency of less than 5% were constipation, sleep disorders, speech disturbances, tiredness, falls and abnormal weight loss. In a sensitivity analysis, excluding pain, the most common first symptoms were vertigo (29.6%), cough (13.6%), urinary disturbances (12.3%), gait abnormalities (10%) and falls (8.6%) and the median time to first diagnosis was 3.6 years (IQR: 2.5–4.6 years). During the 5 years preceding the PSP diagnosis, 85.2% of patients reported that they experienced pain, falls and vertigo were reported both by 59.1% of patients and gait abnormalities were reported by 55.7% of patients (Table 2). The most common misdiagnoses were Parkinson’s disease (PD) and cognitive disorder (both with 51%), depression (50%), Alzheimer’s disease (AD) and dementia (both with 19%). Three and a half percent of patients had a misdiagnosis of convergence insufficiency palsy. The most common new comorbidities are presented in Table 3. Post index, hypertension (10.2%), cerebrovascular disease (10.2%) and cataract (10.2%) were the most common new comorbidities. Thirty-six percent of patients had become disabled prior to the index date. Of those who were un-disabled at the index date, 51% became disabled during the follow-up period. Median time to disability was 2 years post index (95% CI 1.6–2.4) (Figure 4). Treatment patterns are shown in Table S1 and Table 4. Anti-Parkinsonian medications were used by 57% of the patients in the last year prior to the index date and 76% of patients used them in the first year post index date. Of the Anti-Parkinsonian medications, 43% and 60% of patients used levodopa-carbidopa, prior and post index, respectively. The second highest most commonly used generic ingredient was amantadine 20.5% prior index and 27% post index.

The proportions may add up to more than 100% due to multiple comorbidities per patient.

The table shows the proportion of patients that purchased at least one type of medication in the last year prior to the index date and the first year post index date. The proportions may add up to more than 100% due to multiple usage of medication per patient.

## 4. Discussion

This real-world descriptive analysis, to the best of our knowledge, is the first in Israel and one of few in the world [2] to delineate PSP’s epidemiology and its clinical features.

The present study estimated the 2018 crude prevalence rate as 5.3 per 100,000 MHS members over the age of 40. Our results are in line with those published by a recent meta-analysis, reporting a pooled prevalence rate of 7.1 (5.2–9.0), using data from the three studies with appropriate methods [2]. In the literature, prevalence rates vary greatly from 1.39 to 18.1 per 100,000 persons [23,24,25,26,27,28,29]. Nonetheless, for an aged restricted population over 55 years, a prevalence rate of 7 per 100,000 was reported [25]. Discrepancies in the prevalence rates might be a result of various disease definitions (probable vs. possible PSP [26]) of the diversity in case-assignment methods or the awareness and identification of the various disease’s phenotypes.

As in previous reports, PSP prevalence has also been shown to increase with age in this analysis [4,21,28]. Up until the age of 70–74 years’, the prevalence rates are similar to recent reports. However, beyond that age, when the numbers of cases and population decrease, there are unstable rates. In the older age groups (i.e., 75–79 years and 80–84 years), while Viscidi et al. (2021) [4] reported higher prevalence rates (35 and 29 per 100,000, respectively) and Coyle-Glichrist et al. (2018) [21] reported lower rates (11 and 6 per 100,000, respectively), the current analysis shows in-between rates (20 and 18 per 100,000, respectively), increasing this database validity.

The estimated incidence rate of PSP in the current analysis is in line with most previous reports [3,28,30,31], where the rates vary from 0.9 [30] to 2.6 per 100,000 persons per year [3]. Higher rates were suggested in an earlier study, showing a rate as high as 5.3 per 100,000 [31] among people age 50 years or older. In the current study, PSP was first diagnosed at the mean age of 72 years old (SD = 8 years), similar to many previous studies [4,27,29,31]. Nevertheless, some studies show an earlier age of diagnosis [23,25]. Furthermore, the current analysis did not find any sex predominance, in correspondence with previous reports [4,21,23,31].

PSP is a uniformly fatal disease and the survival time ranges between 3 [3] and 10 [25] years, although most studies a report survival time of approximately 5 years [23,29,31]. The present study found a median survival time of 4.9 years. The wide range of survival time might result from various definitions for survival time. Some studies began tracking survival time from the onset of symptoms, while some (including the present study) divided this time into two separate periods, i.e., (1) from symptom onset to diagnosis, and (2) from first diagnosis to death, thus tracking survival time from PSP’s first diagnosis. Another reason for the discrepancy is late diagnosis for the less common PSP phenotypes or less familiar symptoms. Lastly, it may result from an actual difference of survival times between the various phenotypes, such as PSP-RS vs. non-RS phenotypes [7,10]. In the current study, 19.8% (95% CI: 13.3–27.8%) of patients with PSP had a disease duration of more than 10 years (referred as “benign PSP”). These results are in line with a recently published study [32], which found that ocular motor abnormalities within 3 years from disease onset were the only significant independent clinical predictor of long survival (we were not able to support this finding in the frame of our study). Further investigation of patients with benign PSP is warranted.

In the current analysis, for the majority of patients (51%), pain was documented as their first symptom of PSP. After excluding pain, due to its low specificity, the most common symptom was vertigo, presenting in approximately 30% of patients. The term “vertigo”, which is a symptom that arises from the vestibular system, is often used by patients to describe non-vestibular dizziness, which can comprise a sensation of light-headedness, giddiness, unsteadiness, drowsiness or impeding faint [33]. These symptoms might have been incorrectly reported by patients or documented by physicians in lieu of imbalance, a well-established, prominent and early symptom of PSP [34,35,36]. Furthermore, symptoms such as gait abnormalities or falls might be a result of previous undocumented imbalance. Therefore, by combining these results, the proportions of patients who were identified with having vertigo, gait abnormalities and falls may provide us with the true figure of PwPSP who present with imbalance as their first symptom, which, in this case, accounts for almost 50% of the patients.

PSP’s nonspecific early presentation and the absence of a practical, early diagnostic test results in a delayed diagnosis by 3 to 4 years [21,25,29,37]. In accordance with these findings, this study found a gap of 4.2 years between initial symptoms and first PSP diagnosis. This lag shortens to 3.6 years when excluding pain as a symptom. During this period, patients received various misdiagnoses. The most common were PD (51% of this analysis’ patients), AD/dementia/cognitive disorders (almost 90% of patients) and depression (50% of patients). Previous studies support these findings, with PD documented in almost 55% of PwPSP [4,27,38]; memory loss, dementia or AD was found in 70% of patients [4] and depression was reported in approximately 30% of patients [4,38].

This study has shown an increased incidence of cerebrovascular disease in PwPSP after the first PSP diagnosis. This increase might be related to the increased age of the patients. Furthermore, controversial data exist regarding the association between vascular risk factors, especially hypertension and PSP [39,40,41,42,43].

Most patients in the current cohort were treated with anti-Parkinsonian, anti-dementia and anti-depressive medications. The proportion of patients treated with these medication groups increased post diagnosis (57% prior, 76% post; 17% prior, 22% post; and 58% prior, 65% post, respectively). Patients were also treated with anti-vertigo medications (such as betahistine and cinnarizine) in the year prior to first diagnosis and this showed a significant decrease in their post diagnosis consumptions (20% prior, 8% post). These findings correspond with the knowledge of PSP’s symptoms and the symptomatic therapeutic approach. For PSP’s motor symptoms, levodopa is generally attempted, with typically modest to no reaction in most PSP phenotypes, but modest benefit in the PSP-P syndrome [8,11]. Dementia was described as a PSP symptom in the original description of the disease by John Clifford Richardson, John Steele and Jerzy Olszewski in 1963 [44]. Acetylcholinesterase inhibitors are used off-label for cognitive symptoms in PwPSP [11]. The same is true for anti-depressants in PwPSP [11], except for amitriptyline, which was shown to improve motor function in small retrospective PSP cohorts [11].

Unexpectedly, only a small portion (8.6% of those with smoking data) of PwPSP were “ever smokers” (current plus past), contrary to previous publications that reported smoking in approximately 50% of patients [45,46]. However, this number is in line with the proportion of smokers in the elderly population (+65) in Israel [47].

This study has several limitations. This study’s major limitation is the inability to differentiate between disease subtypes. The MHS electronic medical record has existed since the mid-1990’s and uses the ICD-9 codes, as well as an internal, more granulated, coding system. In light of this, PSP’s subtypes may be diagnosed in the clinic but not documented as structured data. For future studies and day to day use, an update of the diagnosis codes to include all PSP’s subtypes according to the 2017 MDS guidelines is warranted [9]. However, looking back to earlier years (as in this study: 2000–2018), subtype stratification is not possible. According to a recently published systematic review and meta-analysis [2], only one of the sixteen identified studies addressed the issue of PSP’s subtypes. Hopefully, in the future, more and more studies based on the relatively new guidelines will be carried out. Second, the requirement of only one PSP diagnosis given by any healthcare professional increases the sensitivity of this study and may decrease specificity. In the clinic, let alone a database scenario, there is a known difficulty in differentiating between atypical parkinsonism, such as PSP, multiple system atrophy and corticobasal degeneration due to overlapping clinical manifestations [48,49]. Nonetheless, the external validity of our study population is supported by previous publications. The prevalence of PSP estimated in our analysis is within the 95% boundaries of the pooled estimate in a recently published meta-analysis [2]. Moreover, our calculated age-specific prevalence of PSP is in line with those previous published by Coyle-Gilchrist [21] et al. and Viscidi et al. [4]. Third, we are not confident that we were able to locate all PSP cases, since the differential diagnosis between PSP and PD is difficult and many cases are diagnosed as Parkinson’s disease or Parkinsonism. Forth, we were not able to analyze the physicians’ summaries in the format of free text; therefore, non-structured symptoms or diagnoses could have been missed. Fifth, this analysis was mostly descriptive with no comparisons to patients without PSP; thus, we are unable to differentiate between actual PSP characteristics (i.e., symptoms, comorbidities, etc.) and age-related characteristics.

## 5. Conclusions

This real-world, database-based study demonstrates that clinic-epidemiological features of PSP in Israel are similar to PSP worldwide. The design of the study did not allow us to perform an analysis according to the PSP phenotypes. However, it may expand the database, which is important for the international collaboration effort in the field of rare neurodegenerative disease research and creating a proper platform for upcoming DMT. Nonetheless, future studies should focus on the identification of different phenotypes and their characterizations.

## Figures and Tables

**Table 1:** Baseline characteristics.

		PwPSP (n = 88)
Age at index	Mean (SD)	72.6 (8.4)
Sex, female	Yes (%)	47 (53.4%)
Socioeconomic status	Low (%)	7 (8.0%)
	Medium (%)	30 (34.1%)
	High (%)	46 (52.3%)
	Missing (%)	5 (5.7%)
Smoking status	Current (%)	4 (4.5%)
	Past (%)	3 (3.4%)
	Never (%)	74 (84.1%)
	Missing (%)	7 (8.0%)

PwPSP = patients with PSP; SD = standard deviation.

**Table 2:** Most common symptoms during the 5 years preceding PSP diagnosis.

Symptom	n (%)
Pain	75 (85.2%)
Fall	52 (59.1%)
Vertigo	52 (59.1%)
Gait abnormalities	49 (55.7%)
Tiredness	31 (35.2%)
Cough	30 (34.1%)
Constipation	21 (23.9%)
Urinary disturbance	21 (23.9%)
Speech disturbance	17 (19.3%)
Abnormality weight loss	15 (17.0%)
Sleep disturbance	12 (13.6%)
Dysphagia	7 (8.0%)
Apraxia	2 (2.3%)

**Table 3:** New diagnoses of most common comorbidities prior and post PSP first diagnosis (ever).

	Diagnosis Prior PSP	Diagnosis Post PSP
Hypertension	59 (67.0%)	9 (10.2%)
Cerebrovascular disease	3 (3.4%)	9 (10.2%)
Cataract	36 (40.9%)	9 (10.2%)
Hypercholesterolemia	24 (27.3%)	7 (8%)
Chronic kidney Disease	53 (60.2%)	6 (6.8%)
Ischemic heart disease	10 (11.4%)	4 (4.5%)
Diabetes	18 (20.5%)	3 (3.4%)
Benign prostatic hyperplasia	21 (23.9%)	3 (3.4%)
Cancer	21 (23.9%)	2 (2.3%)

**Table 4:** Treatment patterns of anti-Parkinsonian generic ingredient.

	One Year Prior Index	One Year Post Index
Levodopa/carbidopa	38 (43.2%)	53 (60.2%)
Amantadine	18 (20.5%)	24 (27.3%)
Levodopa alone	3 (3.4%)	1 (1.1%)
Biperiden	4 (4.5%)	2 (2.3%)
Pramipexole	2 (2.3%)	4 (4.5%)
Rasagiline	3 (3.4%)	5 (5.7%)
Pergolide	2 (2.3%)	1 (1.1%)
Ropinirole	5 (5.7%)	6 (6.8%)
Selegiline hcl	7 (8.0%)	7 (8.0%)
Entacapone	3 (3.4%)	3 (3.4%)

## Data Availability

Data sharing is not applicable. The datasets generated and/or analyzed during the current study are not publicly available because the data that support the findings of this study originate from Maccabi Healthcare Services and restrictions apply to the availability of these data. Because of these restrictions, these data can be accessed only by request to the authors and/or Maccabi Healthcare Services.

## Supplementary Materials

The following supporting information can be downloaded at: https://www.mdpi.com/article/10.3390/brainsci12091126/s1, Table S1: Treatment patterns by pharmacological groups.

## Appendix A. Code Lists

**Table A1:** List of disability codes.

Code	Description
1410	Disability pension
1411	Disability pension over 75% at any age
5550	Home care-chronic patient
5551	Home care-chronic patient
5552	Home care-rehabilitation
5553	Home care-medical
5554	Home care-special medical
5555	Home care-candidate
5556	Home care-home hospice
5557	Home care patient on life support
5558	Home care-assisted living patient on life support
5562	Home care-enhanced
5563	Home hospice recommended
5564	Bedridden patient
5572	Nursing home-assisted living patient
6065	Handicapped parking permit
7678	Social security disability-up to 91%
7679	Social security disability-general
7677	Social security disability-over 91%
8081	Nursing home-assisted living
8082	Nursing home-‘Nativ’ patient
8181	Complex patient
8182	Home hospitalization
8323	Home hospice-demented patient
7675	Social security-foreign worker

**Table A2:** List of symptoms in the analysis.

ICD-9 Code	Symptom
784.69	Apraxia
564	Constipation
786.2	Cough
787.2	Dysphagia
E888	Fall
E888.9	
781.2	Gait abnormality
719.4	Pain
719.46	
724.2	
729.5	
784	
789	
780.5	Sleep disturbance
784.5	Speech disturbance
780.7	Tiredness
788.3	Urinary incontinence
788.41	Urination frequency
780.4	Vertigo
719.7	Walking difficulty
783.2	Weight loss abnormal

**Table A3:** List of misdiagnoses in the analysis.

ICD-9 Code	Misdiagnosis
780.9	Age-related cognitive decline
331.0	Alzheimer’s disease
294.9	Cognitive disorders
378.83	Convergence insufficiency/palsy
290.0	Dementia senile
296.30	Depressive disorders
311	
309.1	Mental distress with physical and behavioral symptoms
332.0	Parkinson’s disease

**Table A4:** List of comorbidities in the analysis (not including registries).

ICD-9 Code	Comorbidity
272.4	Hypercholesterolemia
366.9	Cataract all types
600.9	Benign prostate hyperplasia (BPH)
