# Supplementary material for: Epidemiology of Progressive Supranuclear Palsy: Real World Data from the Second Largest Health Plan in Israel
Source: Brain Sci. 2022 Aug 24;12(9):1126. doi: 10.3390/brainsci12091126 (PMC9496895; doi:10.3390/brainsci12091126)
Supplement: Supplementary file 1 [file brainsci-12-01126-s001.zip › brainsci-1862991-supplementary.pdf]

Supplementary Materials

Table S1: Treatment patterns by pharmacological groups.

	One year prior index	One year post index
Antiparkinson	50 (56.8%)	67 (76.1%)
Analgesic/antipyretic	33 (37.5%)	44 (50.0%)
Gastro-intestinal	41 (46.6%)	48 (54.5%)
Antidepressant	51 (58.0%)	57 (64.8%)
Genital/urinary	30 (34.1%)	36 (40.9%)
Hypnotic/sedative	35 (39.8%)	41 (46.6%)
Antidementia	15 (17.0%)	19 (21.6%)
Laxative	37 (42.0%)	41 (46.6%)
Antithrombotic agents	58 (65.9%)	60 (68.2%)
Antibiotics	44 (50.0%)	46 (52.3%)
Eye preparations	38 (43.2%)	38 (43.2%)
Antidiabetic	15 (17.0%)	15 (17.0%)
Cardiovascular	58 (65.9%)	58 (65.9%)
Respiratory system	12 (13.6%)	12 (13.6%)
Antipsychotic	10 (11.4%)	9 (10.2%)
Hypolipidemic	48 (54.5%)	44 (50.0%)
Antivertigo	18 (20.5%)	7 (8.0%)
